# Forkhead box P3-positive regulatory T cells in immune surveillance and cancer

**DOI:** 10.1038/sj.bjc.6603939

**Published:** 2007-08-14

**Authors:** G Ferretti, A Felici, F Cognettti

**Affiliations:** 1 Division of Medical Oncology A, Regina Elena Cancer Institute, Via Elio Chianesi 53, Rome 00144, Italy

**Sir,**

According to Betts *et al* (2007), immune surveillance would be limited by the inhibitory effect of naturally occurring forkhead box P3 (FOXP3)-positive (+) regulatory T cells. In mice, methylcholanthrene-induced fibrosarcomas were strikingly infiltrated with FOXP3+ regulatory T cells. The partial ablation of FOXP3+ regulatory T-cell activity resulted in a marked reduction in tumour incidence.

Increased frequencies of CD4+CD25^high^ T regulatory (Treg) cells have been registered in the peripheral blood of patients with several types of cancer, suggesting a putative role exerted by these cells in tumour escape from immunological control. There is a significant increased frequency of CD4+CD25^high^ T cells in patients with renal cell carcinoma compared with normal donors (Griffiths *et al*, 2007). These data were confirmed using the FOXP3 marker of Treg cells in a subset of these patients and normal donors. The early follow-up data showed an association between higher peripheral blood Treg cells count and adverse overall survival (Griffiths *et al*, 2007). Similarly, an expansion of increase of FOXP3+CD4+CD25^high^ Treg cells in peripheral blood and tumour microenvironment has been demonstrated in nasopharyngeal carcinoma (NPC) patients (Lau *et al*, 2007). The expanded Treg in the circulation also showed enhanced suppressive activity on CD4+CD25− T-cell proliferations. The increase of this functional Treg population would be able to reduce T-cell-mediated antitumour immunity, since a significant decrease in CD4+ T-cell populations in NPC patients was observed (Lau *et al*, 2007).

Ipilimumab, a fully human anticytotoxic T lymphocyte-associated antigen-4 (CTLA-4) monoclonal antibody, depresses T regulatory (Treg) cell numbers without increasing vaccine-specific CD8+ T-cell responses in patients previously treated with investigational anticancer vaccines (O'Mahony *et al*, 2007). Treg cells, detected by expression of CD4+CD25+CD62L+, decreased at early time points after ipilimumab administration, with a rebound increase by the time of the next treatment. Tumour responses were limited to two patients with non-Hodgkin's lymphoma who experienced limited tumour regression at selected metastatic sites. In one of these patient responders, reverse transcription–polymerase chain reaction for FOXP3 mRNA expression among peripheral blood mononuclear cells showed that FOXP3 expression declined at early time points after ipilimumab administration and rebounded to baseline values by the time of the next infusion (O'Mahony *et al*, 2007).

CD4+CD25+FOXP3+ Treg cells are involved in the maintenance of suppressive control of aberrant immune responses. Mutations in FOXP3 cause multi-organ autoimmunity in both human and mouse (Marson *et al*, 2007). CD4+CD25+FOXP3+ Tregs may impede the development of effective immunity to autologous tumour cells (Yamaguchi and Sakaguchi, 2006), since self-reactive T cells are continuously suppressed by Treg cells. When suppression is relieved, self-reactive T cells become activated and facilitate accelerated maturation of dendritic cells (Kim *et al*, 2007). In fact, the immunisation of mice against FOXP3 elicits a robust FOXP3-specific CTL response, enhancing vaccine-induced antitumour immunity (Nair *et al*, 2007). The combined CD4+CD25+ regulatory T-cell inactivation and genetic vaccination resulted in significant tumour protection in a metastatic tumour model (Elia *et al*, 2007). In another murine model, it has been demonstrated that combining denileukin diftitox, a fusion protein of interleukin-2 and diphtheria toxin, with a vaccine enhances antigen-specific T-cell immune responses (Litzinger *et al*, 2007).

Molecules upregulated on the surface of Treg cells, such as CTLA-4 and CD25, are not expressed exclusively on Tregs (Fontenot and Rudensky, 2004, 2005; Sakaguchi, 2004). The effectiveness of targeting CD25 to eliminate Treg is limited by the fact that CD25, the low-affinity interleukin-2 receptor, is upregulated on conventional antigen-activated T cells (Fontenot and Rudensky, 2004). The only gene product known to be exclusively expressed in Tregs of mice is FOXP3 (Fontenot and Rudensky, 2004). On the other hand, even though FOXP3 expression was initially thought to be restricted to the CD4+CD25+ regulatory T-cell population, recent studies in B-cell non-Hodgkin's lymphoma showed that a subset of intratumoural but not peripheral CD4+CD25− T cells express FOXP3 and are capable of suppressing the proliferation of autologous infiltrating CD8+ T cells (Yang *et al*, 2007).

Nevertheless, based on the findings reported by Betts *et al* (2007), FOXP3-positive Treg cells could represent an important therapeutic target for cancer. The results of this study suggest a role for Tregs in suppressing effective immune surveillance of carcinogen-induced tumours in intact animals. Moreover, besides the enhanced antitumour immunity, the FOXP3 vaccination could lead to the preferential depletion of intratumoural but not peripheral Treg, eventually reducing the risk of autoimmunity (Nair *et al*, 2007). However, further studies are needed to better clarify the weight of Treg depletion within the global immune response to the tumour.
